# Neuroprotective Effect of Polyvalent Immunoglobulins on Mouse Models of Chemotherapy-Induced Peripheral Neuropathy

**DOI:** 10.3390/pharmaceutics16010139

**Published:** 2024-01-20

**Authors:** Mohamad Mroué, Flavien Bessaguet, Angélique Nizou, Laurence Richard, Franck Sturtz, Laurent Magy, Sylvie Bourthoumieu, Aurore Danigo, Claire Demiot

**Affiliations:** 1 UR 20218—NeurIT, Faculties of Medicine and Pharmacy, University of Limoges, 87025 Limoges, France; mohamad.mroue@unilim.fr (M.M.); angelique.nizou@unilim.fr (A.N.); laurence.richard@unilim.fr (L.R.); franck.sturtz@unilim.fr (F.S.); laurent.magy@unilim.fr (L.M.); sylvie.bourthoumieu@unilim.fr (S.B.); aurore.danigo@unilim.fr (A.D.); 2 UMR INSERM 1083 CNRS 6015 MITOVASC Laboratory, CarMe Team, University of Angers, 49045 Angers, France; flavien.bessaguet@univ-angers.fr; 3 Department of Neurology, Reference Center for Rare Peripheral Neuropathies, University Hospital of Limoges, 87042 Limoges, France; 4 Department of Pathology, University Hospital of Limoges, 87042 Limoges, France; 5 Department of Biochemistry and Molecular Genetics, University Hospital of Limoges, 87042 Limoges, France; 6 Department of Cytogenetic, Medical Genetic and Reproductive Biology, University Hospital of Limoges, 87042 Limoges, France; 7 Transversal and Territorial Therapeutic Education Unit (UTTEP87), University Hospital of Limoges, 87042 Limoges, France

**Keywords:** chemotherapy, intravenous immunoglobulins, IVIG, neuropathic pain, cancer, CIPN, mouse models, allodynia

## Abstract

The occurrence of neuropathic pain in chemotherapy-induced peripheral neuropathy (CIPN) is a major dose-limiting effect of many commonly-used anticancer agents. Polyvalent human immunoglobulins (hIg), used in the treatment of several peripheral neuropathies, may alleviate neuropathic pain. The aim of this project was to investigate the preventive effect of hIg in two mouse models of CIPN, induced by vincristine (VCR, 100 µg/kg/d) and oxaliplatin (OXP, 6 mg/kg/3d). Human Ig were administered one day before the first injection of chemotherapy. The onset of CIPN and effects of hIg were assessed via functional tests and morphological analyses of sensory nerves. To evaluate the effect of hIg on chemotherapy cytotoxicity, viability assays were performed using hIg (0 to 12 mg/mL) combined with anticancer agents on human cancer cell lines. The preventive treatment with hIg alleviated tactile hypersensitivity and nerve injuries induced by VCR. It also alleviated tactile/cold hypersensitivities and nerve injuries induced by OXP. Treatment with hIg did not affect the cytotoxicity of either chemotherapy. Furthermore, in combination with VCR, hIg potentiated chemo-induced cell death. In conclusion, hIg is a promising therapy to prevent the onset of CIPN and potentiate chemotherapy effect on cancer, reinforcing the interest in hIg in the management of CIPN.

## 1. Introduction

The occurrence of peripheral neuropathy is a major dose-limiting effect for many commonly-used chemotherapeutic agents, and it greatly affects patient quality of life. A meta-analysis of over 4000 patients receiving chemotherapy revealed that the prevalence of chemotherapy-induced peripheral neuropathy (CIPN) is around 68% during the first month post-treatment [1]. CIPNs are mainly length-dependent, and they are predominantly sensitive polyneuropathies that are either reversible or may persist for several years after cessation of the treatment. The most debilitating symptom is neuropathic pain which can have a particularly negative impact on daily activities, and it represents one of the main causes of impaired quality of life in cancer survivors [2]. In addition, more than half of patients with neuropathic pain develop mood disorders such as depression and anxiety, which are associated with a less favorable prognosis [3,4]. Thus, preventing the onset of CIPN and its progression through an appropriate therapeutic approach is a key priority for maintaining anticancer treatments and for improving patient quality of life during and after the end of chemotherapy.

The pathophysiology is dependent on the agent, and the underlying molecular mechanisms remain unknown. However, an emerging concept concerns the involvement of immune-mediated mechanisms. At first, the injection of chemotherapeutic agents has an immunosuppressive effect due to their cytotoxic effects on immune cells, although, increasing evidence suggests that neurotoxic chemotherapy likely stimulates inflammatory mechanisms that are responsible for CIPN [5]. An increase in cytokine levels, the activation of cytotoxic cells, and the recruitment of macrophages have all been described as being initiated by chemotherapeutic agents such as vincristine (VCR) and oxaliplatin (OXP) [6,7,8,9]. Moreover, nerve lesions attract immune cells, which infiltrate the site of injury and could contribute to the maintenance of pain [10,11]. Recently, a meta-analysis showed that there may be a therapeutic advantage to using polyvalent human immunoglobulins (hIg) to alleviate pain caused by peripheral neuropathies. The results showed that 65% of patients with neuropathic pain who received hIg reported pain relief [12]. Moreover, hIg has been found to have anti-inflammatory effects by regulating cytokine levels, modulating the activity and number of immune cells, and by affecting NFκB levels [13]. In addition, hIg is already used as a first-line therapy in the treatment of peripheral neuropathies such as Guillain–Barré syndrome, chronic inflammatory demyelinating polyneuropathy, and multifocal motor neuropathy, and it is a well-tolerated treatment [14]. Therefore, we believe that hIg could be a promising therapy to prevent CIPN. Preclinical studies have already described the neuroprotective effect of hIg in rat models of paclitaxel- and bortezomib-induced peripheral neuropathies [15,16]. Our goal, in this study, was to assess the neuroprotective effect of hIg on mouse models of VCR- and OXP-induced neuropathy, as well as to examine any impact of hIg on the cytotoxic effects of chemotherapy agents in human cell lines.

## 2. Materials and Methods

### 2.1. Chemicals

The anticancer agents, VCR (vincristine Hospira^®^, 2 mg/2 mL, Pfizer, New York, NY, USA) and OXP (oxaliplatin Hospira^®^, 200 mg/40 mL, Pfizer, New York, NY, USA), were obtained from the Hospital Pharmacy of Limoges and diluted in a physiological solution (NaCl 0.9% in water for injection) for VCR, or in glucose 5% for OXP for animal experiments. Normal human immunoglobulins (hIg) were supplied by CSL Behring France (Privigen^®^, 100 mg/mL, Paris, France) and diluted in saline solution.

### 2.2. In Vivo Studies

#### 2.2.1. Animals

This study was conducted in accordance with the guidelines for the ethical care of experimental animals of the European Community (2010/63/EU), and it was submitted to the French Ministry of Higher Education and Research and approved (number APAFIS#29436-2021020115141016 v1, the 23 February 2021). Animal experiments are reported in compliance with ARRIVE guidelines. A total of 80 male and female (sex ratio = 1:1) Swiss mice (6–7 weeks old) from Janvier labs (Saint Berthevin, France) were housed in groups of 4–5 per cage, and maintained on a 12 h light/dark cycle with food and water available ad libitum (BISCEm-animal care and facility center, Limoges, France). An acclimatization period of 7 days was respected between the introduction into the animal facility and the start of the experiments. Shredded paper nesting material was supplied for environmental enrichment.

#### 2.2.2. Timeline of Treatments

**Figure 1:**
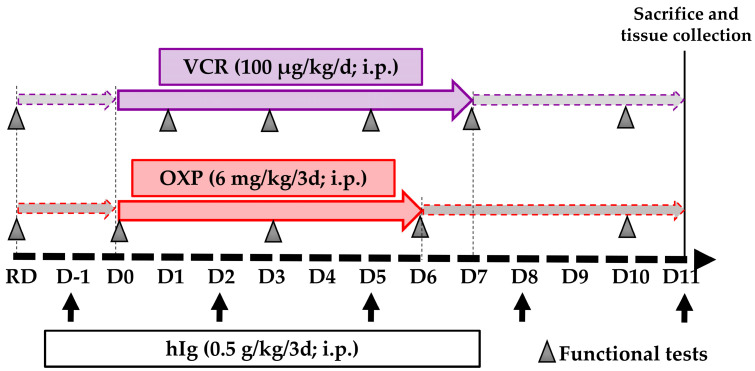
Schematic representation of study design. D: day, hIg: human immunoglobulin, i.p.: intraperitoneal, OXP: oxaliplatin, RD: reference day, VCR: vincristine. Black arrows correspond with hIg administration.

Mouse models of CIPN were established by intraperitoneal injections as detailed below (Figure 1):

- VCR-induced peripheral neuropathy; one daily injection of VCR (100 µg/kg) every day over 7 days [17].
- OXP-induced peripheral neuropathy; one daily injection of OXP (6 mg/kg), every three days over 6 days [18].

Mice in the respective control groups (Ctrl) received an equivalent volume of physiological solution or glucose 5% following the same schedule.

Preventive treatments with hIg (0.5 g/kg) started one day prior to the first chemotherapy administration, and they were administered every 3 days until the end of the experiment (Figure 1). A pharmacokinetic study performed by the hIg supplier (unpublished data) show that the hIg plasma level was maintained at 3 g/L until the third day after an intraperitoneal injection at 0.4 g/kg and the maximal plasmatic concentration was reached at 24 h. Thus, hIg therapy began 24 h before the chemotherapy injection to take this delay into account. This same pharmacokinetic study compared intravenous and intraperitoneal injections in mice, showing that the plasmatic concentration at 24 h was the same with both methods.

Mice without hIg received an equivalent volume of physiological solution. Injections of hIg were performed several hours prior to anticancer agent administration on the days of co-administration. Thus, four groups were created for each anticancer agent, as follows: Ctrl, Chemotherapy, hIg-Ctrl, and hIg-Chemotherapy. Hence, there was a total of 8 groups (n = 10 in each group, sex ratio 1:1). Mice were randomly assigned to each group using an online randomization tool (http://www.graphpad.com/quickcalcs/index.cfm, accessed on 6 April 2021).

An assessment of tactile sensitivity was performed using the von Frey filament test for both CIPN models. A cold plate test (4 °C) was used in the OXP-induced peripheral neuropathy model to evaluate cold nociception. The behavioral tests were assessed by the same researcher. At the end of the experiments, paw skin, dorsal root ganglia (DRG), and the sciatic nerve were then quickly removed for subsequent immunofluorescence and ultrastructural analyses. Throughout the randomization and histological analyses, treatment groups were coded. Thus, behavioral and counting experiments were performed by assessors who were unaware of the treatment (hIg) and the conditions used (Ctrl or Chemotherapy).

#### 2.2.3. Functional Assessments

##### von Frey Filament Test

Tactile sensitivity was assessed using von Frey filaments (Bioseb, Vitrolles, France). The von Frey filament test represents the reference test for the evaluation of the mechanical sensitivity in animals and humans [19]. Mice were placed in a plastic cage with a wire mesh floor which allowed access to the paws. There was an acclimatization period of 1 h before the start of the experiments. The area tested was the mid-plantar left hind paw. A test round started with filament #6 (0.40 g). Depending on the animal’s response, the higher or lower force filament was then applied to obtain the smallest mechanical pressure, which induced a painful response from the animal. Each animal went through three test rounds for each paw under each experimental condition. A test session consisted of 3 successive trials spaced 5 min apart. An average of the 3 trials was calculated, and it corresponded with the animal’s tactile sensitivity threshold in the same session.

#### Cold Plate Test

Thermal nociception was assessed using the cold plate test (Bioseb, Vitrolles, France). Mice were placed on a 4 °C plate to assess sensitivity to the cold [20]. Latency (s) until the first withdrawal criteria was recorded. The criteria of withdrawal included stretching, writhing, freezing, or jumping on the cold plate. A test session consisted of 3 successive trials spaced 5 min apart. An average of the 3 trials was calculated, and it corresponded with the latency of the animal for the same session. Mean latency was expressed as the threshold of an individual animal to cold stimulation.

#### 2.2.4. Quantification of Intra-Epidermal Nerve Fiber (IENF) and DRG Neuron Densities

Animals (n = 6–11 per group) were euthanized through cervical dislocation following isoflurane anesthesia. Subsequently, footpads were excised using a 3 mm punch biopsy, fixed overnight in 4% PFA, cryoprotected with 30% sucrose, and frozen at −20 °C. Sections were sliced at a thickness of 20 µm using a cryostat, and incubated overnight with a primary antibody anti-Protein gene product 9.5 (1:50, PGP9.5, rabbit monoclonal; Abcam, Cambridge, UK). Then, sections were treated with a secondary antibody conjugated with Cy3 (1:500; Jackson Immunoresearch, Suffolk, UK). Epidermal nerve fibers were quantified using an unbiased observer at 400× magnification (Eclipse 50i, Nikon Europe B.V., Amstelveen, The Netherlands), adhering to established guidelines for human studies [21]. The length of the dermo-epidermal junction was determined and defined as the epidermal length using NIS-Elements BR 2.30 software (Laboratory Imaging s.r.o, Nikon, corporation, Prague, Czech Republic). IENF density was determined as the ratio of the number of epidermal nerves to the epidermal length (IENF number/mm). The counting was performed on three slides per mouse.

To evaluate the density of DRG neurons, four lumbar (L4–L5) DRG per mouse were removed and processed as described above, with the exception of a section that was 8 µm thick. Each DRG section was captured at 200× magnification, using fluorescence microscopy, in a systematic manner. PGP9.5^+^ neurons were counted, and only the region containing neurons was measured using NIS-Elements BR2.30 software. The DRG neuron density was expressed as neurons/mm^2^. Three sections per DRG were counted.

#### 2.2.5. Sciatic Nerve Ultrastructural Analysis

Sciatic nerves were dissected and immersed in a 2.5% glutaraldehyde solution diluted in Sorensen’s buffer, then, the nerves underwent dehydration and were embedded in Epon 812 resin (Euromedex, Souffelweyersheim, France). Ultrathin sections were stained with uranyl acetate and lead citrate before being examined using an electron microscope (JEM-1400 Flash, Jeol, Peabody, MA, USA). A total of six photographs per animal (covering the entire section of sciatic nerve) were captured at a magnification of 3000×. The number of myelinated fibers per mm^2^ was then counted to determine the fiber density.

### 2.3. In Vitro Studies

#### 2.3.1. Cell Lines

Four human cancer cell lines were used, comprising two different cell lines that are sensitive to each of the anticancer agents used, as follows: VCR or OXP. The sensitivity of each cancer cell line to chemotherapy agents was based on the literature, the DepMap, and the “Genomics of drugs and sensitivity of cancers” (GDSC) databases. The cell lines used are as follows:

- Two diffuse large B-cell lymphoma cell lines, SU-DHL-4 (ATCC^®^ CRL-2957™, ATCC, Manassas, VA, USA) and U-2932 (DSMZ^®^ ACC633), sensitive to VCR (suspension cell culture).
- Two colorectal cancer cell lines, HT29 (ATCC^®^ HTB-38™) and HCT116 (ATCC^®^ CCL-247™), sensitive to OXP (adherent cell culture),

Cell lines were cultured in the medium recommended by ATCC^®^. Culture media were supplemented with 10% heat-inactivated fetal bovine serum, 1% L-glutamine, 1% non-essential amino acids, and 1% penicillin/streptomycin (Gibco, Waltham, MA, USA). All cells were grown at 37 °C in a humidified air atmosphere containing 5% CO_2_.

#### 2.3.2. Treatments

Cells were seeded in 96-well plates at the density needed to obtain 80% of confluence for 24 h (Corning Falcon, Dominique Dutcher, Bernolsheim, France). After 24 h of incubation, cells were treated with hIg at doses ranging from 0 to 12 mg/mL (corresponding with the minimum concentration in patients’ serum after 3 weeks of i.v hIg treatment in clinical studies), in combination with the anticancer agent at the IC50 for 24 h. IC50 designated the concentration of the anticancer agent, where a 50% reduction in absorbance, compared with the control, was observed (Table 1). The solutions of chemotherapies, as well as hIg, were prepared and diluted directly in a culturing medium suitable for lines. The effect of hIg and chemotherapy agents on cell viability was assessed using an MTT (3-(4,5-dimethylthiazol-2-yl)-2,5-diphenyltetrazolium bromide) assay.

#### 2.3.3. Cell Viability Assay

Following a 24 h treatment period, 20 µL of MTT solution (5 mg/mL) was added to each well containing 100 μL of medium. The cells were then incubated for 3 h. The medium was then removed, and 150 μL of DMSO was added to each well to solubilize the formazan crystals. Absorbance was measured at 490 nm using a microplate reader (Lab Systems, MultiskanEX, Waltham, MA, USA). Cell viability was quantified as the percentage of MTT reduction, with the absorbance value of untreated cells designated as 100%. All experiments were conducted in triplicate, and the results are presented as the mean ± standard error of mean (SEM).

### 2.4. Data Analysis

Data were analyzed using GraphPad Prism 8 software and expressed as the mean ± standard error of the mean (SEM). When statistical significance was identified using a two-way repeated method such as ANOVA, or a mixed-effects model statistical method, individual comparisons were subsequently tested using Tukey’s multiple comparison test for the longitudinal follow-up of functional tests. For morphological analyses, a one-way ANOVA test and Tukey’s multiple comparisons test were used to evaluate differences between multiple groups. Significance levels were represented as follows: * *p*-value < 0.05, ** *p* < 0.01, *** *p* < 0.001, **** *p* < 0.0001. In order to reduce the number of animals used, the morphological results of Ctrl and hIg-Ctrl groups were reused for all three protocols, as the treatments for these two groups were the same.

## 3. Results

### 3.1. Effect of Preventive hIg Treatment on the Onset of VCR-Induced Peripheral Neuropathy in Mice

#### 3.1.1. Immunoglobulins and VCR Did Not Alter Weight Gain of Mice

**Figure 2:**
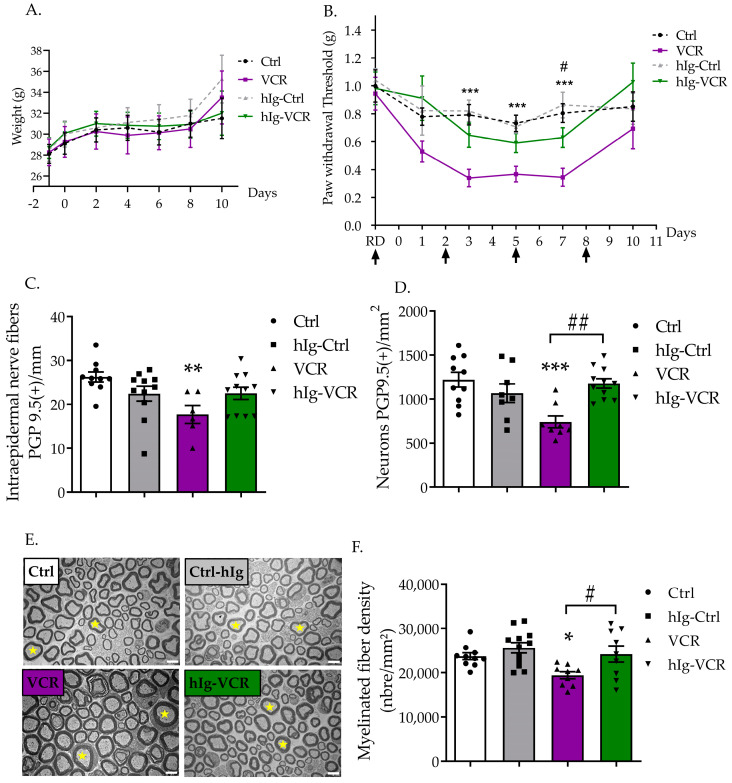
Effect of polyvalent immunoglobulin therapy on a mouse model of vincristine-induced peripheral neuropathy. (**A**) Mouse weight. (**B**) hIg therapy (0.5 g/kg/3d) and VCR effect on mechanical sensitivity was evaluated using the von Frey filament test. Black arrows designate hIg injections. n = 9 to 10 mice per group. (**C**–**F**) Foot pad skin, sciatic nerve, and DRG were removed at D11. Foot pad skin and DRG neurons were immunostained for protein gene product 9.5 (PGP9.5). (**C**) Quantification of PGP9.5-positive intra-epidermal nerve fiber. (**D**) Quantification of DRG neurons positive for PGP9.5. (**E**) Transversal sciatic nerve sections obtained via electron microscopy. Scale bar = 5 µm. Yellow stars indicate myelinated axons. (**F**) Quantification of myelinated nerve fibers in sciatic nerve. Data are expressed as mean ± SEM and they were compared using an ANOVA test followed by Tukey post-test, * *p* < 0.05; ** *p* < 0.01; *** *p* < 0.001 VCR vs. Ctrl; # *p* < 0.05, ## *p* < 0.01 VCR vs. hIg-VCR. hIg: human immunoglobulins, RD: reference day (functional test was performed before hIg injection), VCR: vincristine.

The growth curves for animals in all groups showed an increase over time (time: F(1.22, 39.96) = 35.46), *p* < 0.0001). Neither the hIg treatment, nor VCR, nor the combination of both treatments, influenced weight gain of the mice (treatment: F(3, 36) = 0.09654), *p* = 0.9614, Figure 2A).

#### 3.1.2. hIg Alleviated Tactile Hypersensitivity Induced by VCR

Tactile sensitivity was assessed using the von Frey test. Treatments significantly influenced tactile sensitivity (treatment: F(2, 41) = 12.92, *p* < 0.0001). hIg had no significant effect on tactile sensitivity in Ctrl mice. Untreated mice exposed to VCR developed a significant and transient tactile allodynia from D3 to D7 (*p* < 0.01, VCR vs. Ctrl group). Immunoglobulin therapy alleviated the intensity of VCR-induced tactile allodynia. Indeed, there was no significant difference between Ctrl and hIg-VCR groups (D3: *p* = 0.46, D5: *p* = 0.50). The effect of hIg was significant at D7 (*p* = 0.0471, hIg-VCR vs. VCR) (Figure 2B).

#### 3.1.3. hIg Alleviated Sensory Nerve Injuries Induced by VCR

Paw skin and DRG sections were stained with PGP9.5 to evaluate densities of IENF and DRG neurons. Treatments significantly influenced IENF (F(3, 34) = 4.123, *p* = 0.0135) and DRG neurons densities (F(3, 33) = 7.354, *p* = 0.0007). hIg treatment did not affect IENF or DRG neuron densities in the Ctrl group (IENF: *p* = 0.54, DRG: *p* = 0.59 Ctrl vs. hIg-Ctrl) (Figure 2C,D). A significant reduction in IENF density was observed in mice exposed to VCR (*p* = 0.0057 Ctrl vs. VCR), whereas hIg treatment in VCR-exposed mice blocked the VCR-induced reduction in IENF density. The difference between VCR and hIg-VCR groups was not significant (*p* = 0.45), and no significant difference was found between the hIg-VCR and Ctrl groups (*p* = 0.88) (Figure 2C). The density of DRG neurons was significantly reduced in the VCR group compared with the Ctrl group (*p* = 0.001), whereas treatment with hIg significantly blocked the reduction induced by VCR (*p* = 0.0018 VCR vs. hIg-VCR) (Figure 2D).

No obvious structural alterations were noticed in the sciatic nerves of mice exposed to hIg, VCR, or a combination of both compared with Ctrl mice. A slight increase in axon circularity was observed in the myelinated axons of sciatic nerves from VCR mice (Figure 2E). Treatments significantly influenced myelinated axon density (F(3, 36) = 4.920, *p* = 0.0058). Quantification highlighted that VCR treatment caused a significant reduction in the density of myelinated nerve fibers (*p* = 0.0432 Ctrl vs. VCR) which was prevented by hIg treatment (*p* = 0.027 hIg-VCR vs. VCR) (Figure 2F).

### 3.2. Effect of Preventive hIg Treatment on the Onset of OXP-Induced Peripheral Neuropathy in Mice

#### 3.2.1. Immunoglobulins and OXP Did Not Alter Weight Gain of Mice

**Figure 3:**
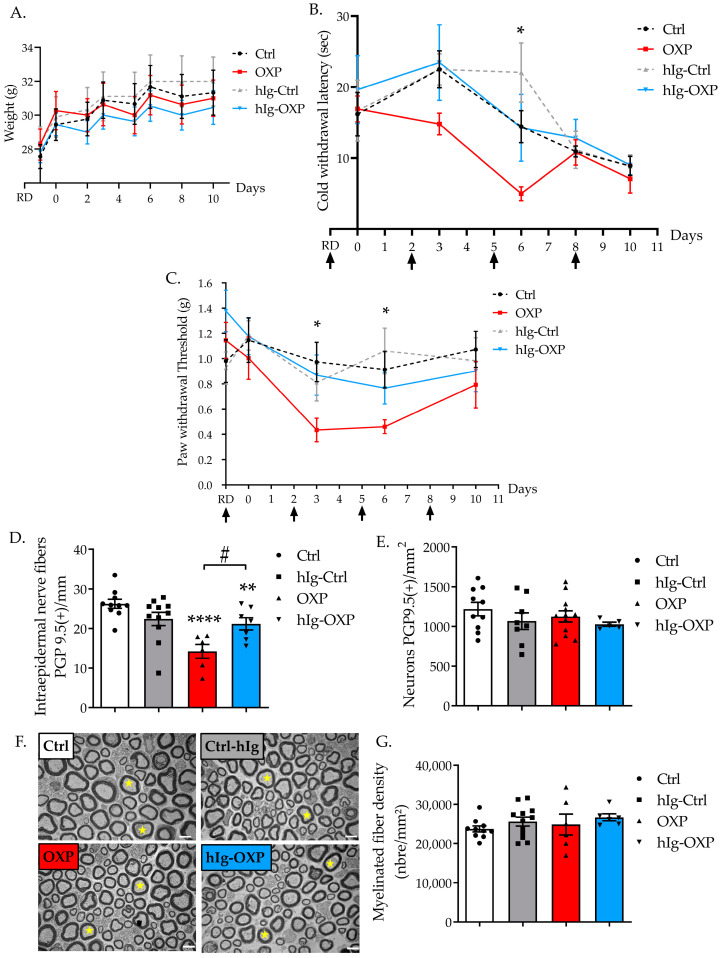
Effect of polyvalent immunoglobulin therapy on a mouse model of oxaliplatin-induced peripheral neuropathy. (**A**) Mouse weight. (**B**) hIg therapy (0.5 g/kg/3d) and OXP effects on mechanical sensitivity, evaluated using the von Frey filament test. Black arrows designate hIg injections. n = 8 to 10 mice per group. (**C**) Cold nociception was evaluated using the cold plate test (4 °C), and each session’s test was performed a few hours after the oxaliplatin injection was administered. Black arrows designate hIg injections. n = 8 to 10 mice per group. (**D**–**G**) Foot pad skin, sciatic nerve, and DRG were removed at D11. (**D**–**E**) Foot pad skin and DRG neurons were immunostained to obtain protein gene product 9.5 (PGP9.5). (**D**) The quantification of intra-epidermal nerve fibers was positive for PGP9.5. (**E**) The quantification of DRG neurons was positive for PGP9.5. (**F**) Transversal sciatic nerve sections were obtained via electron microscopy. Scale bar = 5 µm. Yellow stars indicate myelinated axons. (**G**) Quantification of myelinated nerve fibers in the sciatic nerve. Data are expressed as mean ± SEM and they were compared using an ANOVA test followed by a Tukey post-test, * *p* < 0.05; ** *p* < 0.01; **** *p* < 0.0001 OXP vs. Ctrl; # *p* < 0.05 VCR vs. hIg-OXP. hIg: human immunoglobulins, OXP: oxaliplatin, RD: reference day (the functional test was performed before the hIg injection).

The weights of the animals in all groups increased over time (time: F(3.931, 141.5) = 59.87, *p* < 0.0001). Neither the hIg treatment, nor the OXP, nor the combination of both influenced weight gain (treatment: F(3, 36) = 0.2261), *p* = 0.8776, Figure 3A).

#### 3.2.2. hIg Prevented Cold Hyperalgesia Induced by OXP

Cold hyperalgesia was assessed using the cold plate test (4 °C), two hours after each OXP injection. Indeed, cold hyperalgesia is the hallmark of oxaliplatin-induced acute neurotoxicity, and it develops in patients within hours after the oxaliplatin infusion has been administered [22]. Treatments significantly influenced cold nociception (treatment: F(2, 11) = 3.966, *p* = 0.049). Immunoglobulins did not modify cold nociception in Ctrl mice. OXP administration induced a significant cold hyperalgesia at D6 (*p* = 0.0378, Ctrl vs. OXP group). There was no significant difference between OXP and hIg-OXP groups over time. There was no significant difference between Ctrl and hIg-OXP groups over time. Immunoglobulin therapy tended to prevent OXP-induced cold hyperalgesia (Figure 3B).

#### 3.2.3. hIg Prevented Tactile Allodynia Induced by OXP

Immunoglobulin administration did not affect sensory function when evaluated with the von Frey test. The two-way ANOVA test showed no significative difference between groups (treatment: F(3, 35) = 1.692, *p* = 0.1865). However, the Tukey’s multiple comparisons test showed that OXP induced a significant transient mechanical allodynia in D3 (*p* = 0.0225, OXP vs. Ctrl) to D6 (*p* = 0.0424, OXP vs. Ctrl). There was no significant difference between OXP and hIg-OXP groups over time. There was no significant difference between Ctrl and hIg-OXP groups over time. Immunoglobulins tended to prevent the development of tactile allodynia induced by OXP (Figure 3C).

#### 3.2.4. hIg Prevented Sensory Nerve Injury Induced by OXP

Treatments significantly influenced IENF density (F(3, 30) = 8.859, *p* = 0.0002). hIg treatment did not affect IENF or DRG neuron densities in the Ctrl group (IENF: *p* = 0.50, DRG: *p* = 0.59 Ctrl vs. hIg-Ctrl) (Figure 3D,E). A significant reduction in IENF density was observed in mice exposed to OXP that did not receive hIg treatment (*p* < 0.0001 Ctrl vs. OXP). hIg treatment in OXP-exposed mice significantly mitigated the reduction in IENF density induced by OXP (*p* = 0.0148 hIg-OXP vs. OXP) (Figure 3D). The density of DRG neurons was not affected by OXP exposure (F(3, 31) = 0.8722, *p* = 0.4660, Figure 3E).

No obvious structural alterations were found in the sciatic nerves of mice exposed to hIg, OXP, or the combination of both when compared with the Ctrl mice (Figure 3F). The quantification of myelinated nerve fiber density showed no difference between groups (F(3, 30) = 0.8146, *p* = 0.4959, Figure 3G).

### 3.3. Effect of hIg on the Chemotherapy-Induced Cytotoxicity of Human Cancer Cell Lines

#### 3.3.1. hIg Did Not Alter VCR-Induced Cytotoxicity in Human Lymphoma Cell Lines and Potentiated VCR Cytotoxicity

**Figure 4:**
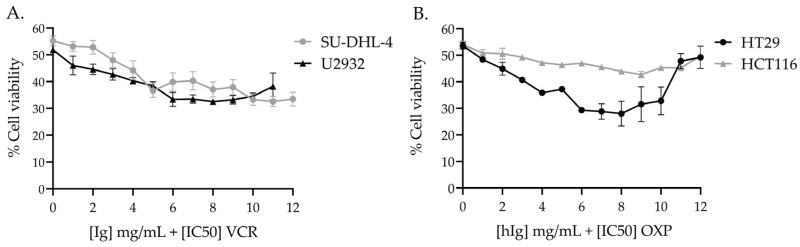
Effect of hIg on the chemotherapy-induced cytotoxicity of human cancer cell lines. (**A**) Effect of the combination of VCR and hIg on the viability of human cancer lines, U2932 and SU-DHL-4. Graph represents three independent experiments (n = 3). (**B**) Effect of the combination of OXP and hIg on the viability of human cancer cell lines, HT-29 and HCT-116. Graph represents three independent experiments (n = 3). Data are expressed as mean ± SEM. hIg: human immunoglobulins, OXP: oxaliplatin, VCR: vincristine.

The concentration of VCR that is required to produce 50% cell viability was 0.95 nM for U2932 cells and 1.06 nM for SU-DHL-4 cells (Table 1). The combination of hIg with VCR at the [IC50] concentration did not negatively interfere with VCR-induced cytotoxicity in either cell line. Moreover, the addition of hIg in combination with VCR reduced cell viability by more than 50%, in a hIg dose-dependent manner (Figure 4A). The addition of hIg enhanced VCR-induced cytotoxicity in U2932 and SU-DHL-4 cell lines by approximately 15%, from 6 mg/mL to 12 mg/mL (Figure 4A).

#### 3.3.2. hIg Did Not Alter OXP-Induced Cytotoxicity in Colorectal Cancer Human Cell Lines

The concentration of OXP needed to produce 50% cell viability was 10.88 µM for the HT29 line and 16.85 µM for the HCT116 line (Table 1). The combination of hIg with OXP at the [IC50] concentration did not negatively interfere with OXP-induced cytotoxicity in either cell line (Figure 4B). At the highest dose (12 mg/mL), hIg did not have an effect on the cytotoxic activity of OXP in HT29 or HCT116.

## 4. Discussion

The main findings of this study are as follows: (1) hIg therapy alleviated painful symptoms in mouse models of VCR-, and OXP-induced peripheral neuropathy; (2) hIg therapy was neuroprotective since treatment improved or prevented nerve injury induced by chemotherapy agents; and (3) the combination of hIg with an anticancer agent did not interfere with chemotherapy-induced cytotoxicity in human cancer cells lines.

In our study, hIg (a preventive treatment) was injected just prior to commencing the chemotherapy protocol, with the aim of preventing the development of neuropathic pain. Such preventive treatments are perfectly applicable in the context of CIPN, since chemotherapy protocols are planned in a hospital setting. Moreover, duloxetine is currently the only recommended drug for the management of CIPN, and it is only moderately recommended. However, duloxetine and other recommended drugs for the treatment of neuropathic pain are used to treat symptoms once the neuropathic pain is established. In accordance with ASCO and ESMO guidelines, no preventive strategy is recommended at present [23,24]. Our protocol evaluates the neuroprotective effect of hIg, a blood-derived product that is already marketed and used for a wide variety of matters. A drug repositioning strategy is relevant in the context of CIPN, since many published drug candidates have failed in clinical trials.

Here, we report that preventive treatment with hIg significantly prevented painful symptoms and nerve injuries induced by chemotherapy in our mouse models of VCR- and OXP-induced peripheral neuropathy. These two molecules are representative of two classes of anticancer agents, vinca-alkaloids and platinum-based drugs, respectively. Moreover, they are among the most neurotoxic agents that are routinely used. Each class of anticancer agent leads to a specific peripheral neuropathy that is characterized by specific symptoms, which are likely to be linked to the anticancer mechanism of action in each class [25]. In addition, some differences were observed between molecules of the same class; for example, the acute cold allodynia was specific to OXP and absent with cisplatin, though both are platinum, salt-derived molecules [26].

Our results are supported by earlier studies which also reported that hIg therapy is neuroprotective for symptoms and nerve injuries induced by paclitaxel and bortezomib in rat models [15,16]. More specifically, hIg therapy improved motor function, reduced thermal hyperalgesia, and preserved the structure of peripheral nerves in rats. Additionally, hIg therapy reduced the accumulation of macrophages and T lymphocytes in DRG; these cells are key components of the immune response in the nervous system [15,16]. A recent study by Tanaka and Kajii (2022) also showed that hIg suppressed painful symptoms of CIPN induced by paclitaxel (in rats and mice) and doxorubicin (in rats) [27]. In our study, we found that hIg was also neuroprotective in CIPN induced by VCR and OXP. Thus, despite the specific neurotoxicity effects of each anticancer agent, hIg seems to be neuroprotective against CIPN, regardless of the class of anticancer agents, in rodent models. Thus, both our results and published data highlight the potential of hIg therapy as a treatment option for preventing CIPN that is induced by a wide range of neurotoxic chemotherapy agents.

Numerous studies have highlighted that traditional anticancer agents, such as taxanes, vinca-alcaloids, platinum derivatives, and proteasome inhibitors, lead to the modulation of some common and specific neuroinflammatory processes involving glial cells (mainly satellite glial cells and Schwann cells), resulting in the onset of sensory impairments and neuropathic pain [7,28,29]. The beneficial effect of hIg on painful symptoms in CIPN rodent models is likely, due to its immunomodulatory effects that may lead to a reduction in neuroinflammation and its associated symptoms. Further studies are now needed to explore the inflammatory state in our CIPN models, in the presence or absence of hIg therapies, in order to answer this question.

It should be noted that hIg has generally been administered to rodents. Employing polyvalent immunoglobulins from the same species would be more appropriate. Indeed, Xu et al. (2019) compared the effects of mouse Ig and human Ig in tumor-bearing BALB/C mice [30], and found that the intravenous hIg injection negatively impacted health status, and caused significant weight loss, compared with mice receiving intravenous mIg; this suggests that mIg is more suitable than hIg in mouse models [30,31]. Though we did not note any changes in the general state of the mice or in mouse weight due to hIg administration, it would be useful to evaluate mIg administration in mouse models of CIPN, in order to better investigate the mechanisms underlying its neuroprotective properties, without the risk of adverse reactions.

Under our experimental conditions, hIg therapy was beneficial for painful symptoms induced by chemotherapy, but it also exhibited neuroprotective effects, with a significant effect observed for nerve injuries (notably the reduction in IENF density). In addition to its immunomodulatory effect, it is potentially responsible for the attenuation of neuroinflammation and the alleviation of pain; we suggest that hIg may have direct neuroprotective effects on the sensory nerves blocking the toxicity induced by anticancer agents. Consistent with this hypothesis, Tzekova et al. demonstrated that hIg could bind directly with Schwann cells via the Fc gamma receptor CD64, and it positively influenced Schwann cell differentiation during culturing, leading to an increase in axonal outgrowth from mouse DRG explants [32]. This shows that hIg plays a crucial role in the regenerative properties of Schwan cells on nerves, and thus, it could directly stimulate nerve protective/regenerative mechanisms.

Taking into account our results and the published data, hIg represents a potential therapy for the management of CIPNs, due to its immunomodulatory and anti-inflammatory effects that could inhibit the neuroinflammation that is common to all CIPNs, as well as its direct neuroprotective properties for nerves. However, in order to prevent the occurrence of CIPNs, neuroprotective molecules, such as hIg, will have to be administered in combination with chemotherapy agents, and not interfere with the anticancer activity of the chemotherapy agent. For this reason, we evaluated the effect of a joint treatment concerning hIg and anticancer agents on human cancer cell line viability. The main finding of these experiments was that hIg did not negatively affect the cytotoxicity of VCR and OXP on human cell lines that are representative of the most frequent cancers treated using these neurotoxic chemotherapies. Indeed, at the [IC50] concentration, both anticancer treatments induced a reduction in cell viability by 50% or less, in combination with a wide range dose of hIg. Under our experimental conditions, hIg treatment does not seem to affect the anticancer effect of OXP in colorectal cancer lines, HT29 and HCT116. However, a recent study reported that hIg treatment reduced the anticancer efficacy of OXP on human colorectal cancer cells [33]. The authors showed that hIg disrupted the ERK1/2 signaling pathway involved in the antitumor activity of OXP. Therefore, our results obtained using cell lines should be noted with caution, and further investigations are needed to assess the impact of hIg therapy on the anticancer properties of OXP. Indeed, this experiment is in its preliminary stages, as only 2D cancer cell lines were used, and only a viability test was performed. Deeper investigations are needed to explore the impact of hIg treatment on the anticancer properties of chemotherapies; for example, patient-derived cancer cells, more physiologic 3D in vitro models, or patient-derived xenograft models could be used [34,35]. This investigation is a prerequisite for setting up future clinical trials to assess the neuroprotective effect of hIg in cancer patients treated with neurotoxic chemotherapy.

However, although research on the role of hIg in cancer treatment is still relatively scarce, some evidence suggests that it may have promising beneficial effects. Several case reports of co-treatments with hIg and chemotherapy highlight a good tolerance of both treatments, and the non-negative impact of hIg on the anticancer activity of chemotherapies [36,37,38,39,40]. Furthermore, clinical and experimental studies have already reported on the potential antitumor and anti-metastatic effects of hIg [30,41,42,43,44,45,46]. Inflammation has been shown to be a critical factor in the growth, progression, and prognosis of cancer [47], and the administration of anti-inflammatory drugs has been shown to lower the occurrence and relapse of different cancers, and it may improve prognosis. Anti-inflammatory agents are thus being progressively used in conjunction with standard cancer treatments, and they are anticipated to provide new therapeutic possibilities for cancer management [47]. In this study, our results reinforce the potential interest of hIg in the management of CIPN and of cancer, through its neuroprotective actions and its beneficial effects on the cytotoxicity of anticancer agents. The safety profile of hIg is very good, with a low occurrence of adverse events; most of them are clinically mild to moderate [48]. As a result, hIg is increasingly considered by clinicians as a “panacea” or a last resort for a growing number of diseases, including anemias, lupus, transplant rejection, and chronic pain, especially when these diseases do not respond to conventional therapies [14]. However, this growing demand exceeds plasma supply, which has led to a global shortage for many years. Some of the most important anti-inflammatory mechanisms of hIg are thought to be dependent of the Fc portion of immunoglobulins [49]. Thus, many recent studies have focused on a novel alternative to hIg in order to overcome the dependence on human plasma supply and mitigate the global shortage issue [50]. These next generation treatments could be the solution which prevents the occurrence of CIPN and its devastating consequences on quality of life in years to come.

## Figures and Tables

**Table 1:** Determination of IC50 for each cell line.

Anticancer Agent	Cell Line	Initial Seeding Density	IC50 Obtained
VCR	U2932	1 × 10^4^ cells	0.95 nM
	SU-DHL-4	2 × 10^4^ cells	1.06 nM
OXP	HT29	0.7 × 10^4^ cells	10.88 µM
	HCT116	0.4 × 10^4^ cells	16.85 µM

## Data Availability

The data presented in this study are available on request from the corresponding author.
